# Healthy CD4^+^ T lymphocytes are not affected by targeted therapies against the PI3K/Akt/mTOR pathway in T-cell acute lymphoblastic leukemia

**DOI:** 10.18632/oncotarget.10984

**Published:** 2016-08-01

**Authors:** Ayman A.M. Alameen, Carolina Simioni, Alberto M. Martelli, Giorgio Zauli, Simona Ultimo, James A. McCubrey, Arianna Gonelli, Giorgia Marisi, Paola Ulivi, Silvano Capitani, Luca M. Neri

**Affiliations:** ^1^ Department of Morphology, Surgery and Experimental Medicine, University of Ferrara, Ferrara, Italy; ^2^ Department of Chemical Pathology, Faculty of Medical Laboratory Sciences, University of Khartoum, Khartoum, Sudan; ^3^ Department of Biomedical and Neuromotor Sciences, University of Bologna, Bologna, Italy; ^4^ Department of Microbiology & Immunology, Brody School of Medicine, East Carolina University, Greenville, NC, USA; ^5^ Biosciences Laboratory, Istituto Scientifico Romagnolo per lo Studio e Cura dei Tumori (IRST) IRCCS, Meldola, Italy; ^6^ LTTA Center, University of Ferrara, Ferrara, Italy

**Keywords:** T lymphocytes, PI3K/Akt/mTOR signaling, T-acute lymphoblastic leukemia, targeted therapies, autophagy

## Abstract

An attractive molecular target for novel anti-cancer therapies is the phosphatidylinositol 3-kinase (PI3K)/Akt/mammalian target of rapamycin (mTOR) pathway which is commonly deregulated in many types of cancer. Nevertheless, the effects of PI3K/Akt/mTOR inhibitors on T lymphocytes, a key component of immune responses, have been seldom explored. In this study we investigated the effects on human CD4^+^ T-cells of a panel of PI3K/Akt/mTOR inhibitors: BGT226, Torin-2, MK-2206, and ZSTK474. We also assessed their efficacy against two acute leukemia T cell lines. T lymphocytes were stimulated with phytohemagglutinin. Inhibitor effects on cell cycle and apoptosis were analyzed by flow cytometry, while cytotoxicity was assessed by MTT assays. In addition, the activation status of the pathway as well as induction of autophagy were analyzed by Western blotting.

Quiescent healthy T lymphocytes were unaffected by the drugs whereas mitogen-stimulated lymphocytes as well as leukemic cell lines displayed a cell cycle block, caspase-dependent apoptosis, and dephosphorylation of key components of the signaling pathway. Autophagy was also induced in proliferating lymphocytes and in JURKAT and MOLT-4 cell lines. When autophagy was inhibited by 3-methyladenine or Bafilomycin A1, drug cytotoxicity was increased, indicating that autophagy is a protective mechanism.

Therefore, our findings suggest that PI3K/Akt/mTOR inhibitors preserve lymphocyte viability. This is a valuable result to be taken into account when selecting drugs for targeted cancer therapy in order to minimize detrimental effects on immune function.

## INTRODUCTION

The phosphatidylinositol-3 kinase/Akt/mTOR (PI3K/Akt/mTOR) signal transduction pathway is activated by several stimuli regulating cell proliferation and survival, translation, autophagy and metabolism. Several members of the PI3K/Akt/mTOR cascade play crucial roles in maintaining cell homeostasis under normal physiological conditions [1].

PI3K comprises a family of lipid kinases which phosphorylate the 3-OH group of inositol lipids. They are classified into Class I, II, and III based on primary structure and regulation. Class I PI3Ks are heterodimeric enzymes, displaying a catalytic and a regulatory subunit. The catalytic subunits comprise p110α, p110β, p110γ, and p110δ, which associate in different ways with the regulatory subunits (p85α, p85β, and p55γ, p101, p84, and p87PIKAP) [2].

Akt requires phosphorylation of both Ser473 and Thr308 amino acidic residues to be fully activated and regulates several downstream, processes including positive activation/regulation of mTOR functions [3].

mTOR is a 289-kDa serine/threonine protein kinase, which regulates cell growth. mTOR is the catalytic subunit of two multi-protein complexes: complex 1 (mTORC1) and mTOR complex 2 (mTORC2).

mTORC1 is responsive towards growth factors, nutrients, energy, or oxidative stress and phosphorylates 4E-BP1 and S6K which are important for protein translation. mTORC2 phosphorylates Akt at the Ser473 residue for complete activation of the PI3K/Akt/mTOR pathway. Other downstream substrates of mTORC2 include PKC [4, 5].

T-cell acute lymphoblastic leukemia (T-ALL) is a malignant disease of T lymphocytes progenitors, characterized by the accumulation of immature undifferentiated thymocytes that acquired multiple genetic aberrations. This results in a poor prognosis, especially for relapsed patients [6]. Aberrant regulation of the PI3K/Akt/mTOR axis often confers a proliferative advantage to tumor cells and contributes to the development of drug-resistance mechanisms.

At present only class I PI3Ks have been shown to be associated with cancer: indeed PIK3CA, the gene which encodes for p110α PI3K, is mutated in a variety of tumor types [7–9]. Mutations of the p85 subunit have also been shown to be oncogenic [10, 11].

The PI3K/Akt/mTOR signaling pathway is frequently up-regulated in T-ALL. This is due to several causes, including phosphorylation, oxidation and gene mutation/deletion, which affect PTEN phosphatase function [12, 13].

Thus, targeting the PI3K/Akt/mTOR axis represents an attractive novel therapeutic strategy for T-ALL. However, which drug against one or more members of this signaling network can achieve the greater efficacy is still an open question.

To address this issue we employed a pharmacological approach aimed to compare the efficacy of four drugs, one targeting mTORC1/C2 (Torin-2) [14, 15], one against PI3K/mTORC1/C2 (NVP-BGT226) [16, 17], one targeting Akt (MK-2206) [18–21], and the fourth one directed against all the class I PI3K isoforms (ZSTK474) [22–24].

Only a limited number of studies have analyzed the impact of PI3K/Akt/mTOR network inhibitors on human T-cells. The few published data have been mainly focused only on PI3K inhibitors, such as wortmannin and LY294002 [25] or pan class I PI3K inhibitors [26] or drugs targeting selectively p110δ [27, 28] or p110α [24].

Drugs targeting the PI3K/Akt/mTOR cascade can interfere with diverse biologic processes also in healthy cells, thus rising concerns about their use in therapeutics. In particular, it is essential to gain knowledge about their effect on immune cells, as it would be desirable to preserve patient's immunity.

The aim of this study was to analyze the effect of the four above mentioned drugs in human primary CD4^+^ T-cells. most of these inhibitors are currently being tested in clinical trials. We have assessed the anti-tumor activity of NVP-BGT226 (BGT226), Torin-2, MK-2206 and ZSTK474 in human T-ALL cells and studied their effects on healthy CD4^+^ T-cell, induced or not to proliferate.

Our findings suggested that, to minimize off-target effects and to benefit from the optimal response, selective inhibitors targeting the PI3K/Akt/mTOR may represent a new promising treatment for T-ALL patients, since they showed a strong cytotoxicity against leukemic T-cells or T-lymphocytes stimulated to proliferate, whereas they did not compromise viability of quiescent healthy CD4^+^ T lymphocytes.

## RESULTS

### Activation status of PI3K/Akt/mTOR pathway in healthy CD4^+^ T lymphocytes and T-ALL cell lines

By Western blot analysis, we wanted to evaluate the baseline levels of some key proteins involved in the PI3K/Akt/mTOR axis in both unstimulated and stimulated healthy CD4^+^ T lymphocytes and in T-ALL cells (MOLT-4 and JURKAT cell lines). We decided to study CD4^+^ cells as they are helper cells that play important roles for regulating immunological responses [29].

**Figure 1:**
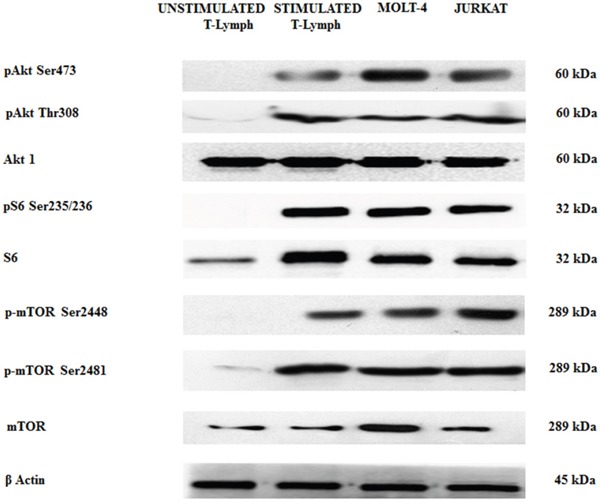
Expression and phosphorylation status of Akt, mTOR and the S6 downstream target in primary (unstimulated and stimulated) CD4^+^ T lymphocytes and T-ALL cell lines Western blot analysis of primary T lymphocytes and T-ALL cell lines to detect the expression and phosphorylation levels of Akt, mTOR, and S6 protein. Twenty-five μg of protein were blotted on each lane. β-actin was revealed as loading control.

Stimulated T lymphocytes, MOLT-4 and JURKAT cells showed a relevant phosphorylation at Ser473 and Thr308 of Akt and at Ser235/236 of ribosomal protein S6 kinase, a readout of mTORC1 activity. The same cell types also displayed mTOR phosphorylation at Ser2448 and Ser2481 residues, readout for mTORC1 and mTORC2, respectively. The phosphorylation was not evident in unstimulated CD4^+^ T lymphocytes (Figure 1).

### BGT226, Torin-2, MK-2206 and ZSTK474 are cytostatic and cytotoxic to stimulated T lymphocytes and T-ALL cell lines

BGT226 is an ATP-competitive dual PI3K/mTORC1/2 inhibitor used for treatment of advanced solid tumors [30, 31]. As previously reported by our group, it was cytotoxic to a panel of hepatocarcinoma cell lines, under both normoxia and hypoxia conditions [32]. Torin-2 potently targets mTORC1/2, and is an effective inhibitor of ATM, ATR and DNA-PK [14].

The efficacy of MK-2206 as well as ZSTK474 have been tested in various preclinical models of human cancers, including leukemias [3, 19, 22, 33–36].

**Figure 2:**
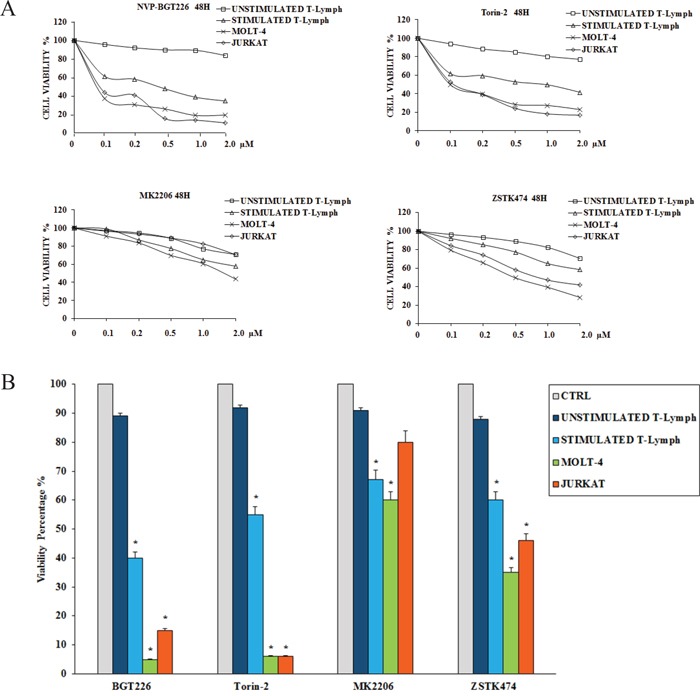
Cytotoxicity of BGT226, Torin-2, MK-2206 and ZSTK474 in primary T lymphocytes, MOLT-4 and JURKAT cell lines **A.** MTT assays of primary T lymphocytes and T-ALL cell lines treated with increasing concentrations of BGT226, Torin-2, MK-2206 and ZSTK474 for 48 h. SD was less than 7%. One representative experiments of three is shown. **B.** Viability of primary T lymphocytes, MOLT-4 and JURKAT cell lines treated for 48 h with 1 μM BGT226, Torin-2, MK-2206 and ZSTK474. Asterisks indicate statistically significant differences with respect to untreated cells (*p<0.05).

To determine how these inhibitors could affect the viability of the primary CD4^+^ T lymphocytes (both unstimulated and stimulated) and T-ALL cells, we performed MTT assays. Cells were incubated for 48 h with the inhibitors, and then cell survival was analyzed (Figure 2A and Table 1). Except for the unstimulated T lymphocytes, BGT226 and the mTORC1/2 inhibitor Torin-2 turned out to be the most powerful drugs in these cells. For unstimulated T lymphocytes, there was no relevant inhibition for both drugs (IC_50_ > 2 μM). For BGT226, cell viability impairment was more evident in T-ALL cell lines, with IC_50_ values of 0.08 μM for JURKAT, 0.06 μM for MOLT-4 and 1.61 μM for stimulated T lymphocytes. Similar results were obtained with Torin-2, with IC_50_ values of 0.1 μM for JURKAT and MOLT-4 and 1.8 μM for stimulated T-cells.

**Table 1:** IC_50_ values of cells treated for 48 hours with different drugs

	BGT226	Torin-2	MK2206	ZSTK474
UNSTIMULATED T-Lymphocytes	>2.0	>2.0	>2.0	>2.0
STIMULATED T-Lymphocytes	1.61	1.8	>2.0	>2.0
MOLT-4	0.06	0.1	1.58	0.52
JURKAT	0.08	0.1	>2.0	0.95

Values are expressed in μM

Regarding MK-2206, the cells displayed higher values of IC_50_ (> 2 μM) except for MOLT-4 cells with an IC_50_ of 1.58 μM. For ZSTK474 the same resistance for the primary T lymphocytes was observed, while in MOLT-4 and JURKAT cell lines the sensitivity of the drug was evident, with IC_50_ values of 0.52 μM and 0.95 μM, respectively. The IC_50_ of stimulated T lymphocytes was > 2 μM (Table 1).

To further assess the cytotoxicity of inhibitors targeting PI3K/Akt/mTOR, we analyzed the changes in cell viability using flow cytometry after treatment with 1μM of each drug for 48 h. Compared to MK-2206 and ZSTK474, BGT226 and Torin-2 displayed higher cytotoxic effect on stimulated T lymphocytes, MOLT-4 and JURKAT cells. None of the drugs affected the viability of unstimulated CD4^+^ T lymphocytes (Figure 2B). For all these reasons, for the subsequent experiments we decided to test only BGT226 and Torin-2.

**Figure 3:**
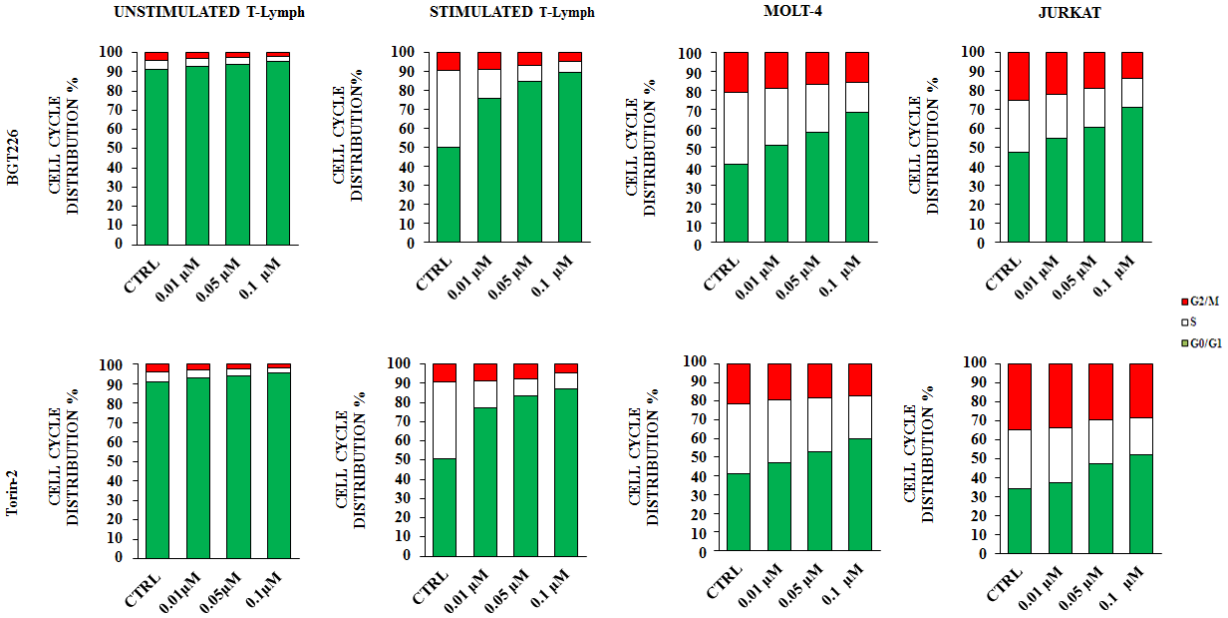
BGT226 and Torin-2 affect cell cycle in primary T lymphocytes, MOLT-4 and JURKAT cell lines Unstimulated and stimulated T lymphocytes, MOLT-4 and JURKAT cells were treated with increasing concentrations of BGT226 and Torin-2 for 24 h. CTRL, control (untreated) cells. SD was less than 10%.

Considering the important role of the PI3K/Akt/mTOR signaling cascade in regulating cell proliferation [37], we investigated the effect of the drugs on cell cycle progression. Cells were treated with the two most effective drugs (BGT226 and Torin-2) for 24 h and stained with Propidium Iodide (PI) for flow cytometric analysis. A concentration dependent increase of cells in the G_0_/G_1_ phase of the cell cycle and a concomitant decrease in cells of both S and G_2_/M phase were observed (Figure 3). The increase was highly significant in stimulated T lymphocytes, MOLT-4 and JURKAT cells, whereas did not occur in unstimulated T lymphocytes.

### BGT226 and TORIN-2 down-regulate the PI3K/Akt/mTOR pathway in stimulated CD4^+^ T lymphocytes and T-ALL cells

**Figure 4:**
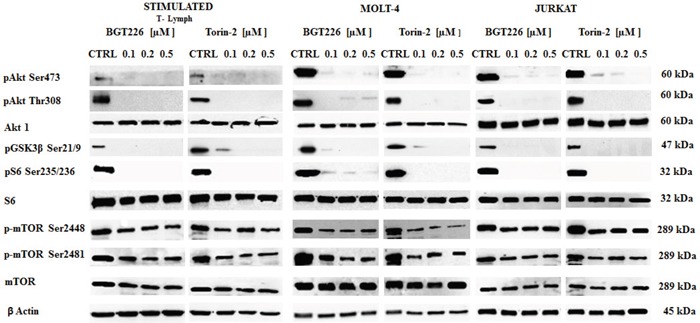
Expression and phosphorylation status of mTOR, Akt and their downstream targets in primary T lymphocytes and T-ALL cell lines Western blot analysis of phosphorylated and total Akt, mTOR and their substrates GSK3β and S6 in stimulated T lymphocytes, MOLT-4 and JURKAT cells treated for 2 h with increasing concentrations of BGT226 and Torin-2. For all experiments twenty-five μg of protein was blotted on each lane. β-actin served as loading control.

To determine whether BGT226 and Torin-2 could affect factors that promote cell survival, stimulated T lymphocytes, MOLT-4 and JURKAT cells were treated with increasing concentrations of BGT226 and Torin-2 for 2 h and then analyzed by Western blot (Figure 4). The inhibition of mTORC2 had a readout in Ser473 Akt dephosphorylation and it was observed in all cell types treated with BGT226 and Torin-2 starting from the lowest concentrations. Thr308 Akt was dephosphorylated by both drugs, as was GSK3β Ser21/9, an Akt substrate. The mTORC1 substrate S6 was completely dephosphorylated on Ser235/236 residue, already at the lowest concentrations of BGT226 and Torin-2. Both drugs downregulated the phosphorylation levels of mTOR at both Ser2448 and Ser2481 residues [38].

### BGT226 and TORIN-2 induce autophagy

**Figure 5:**
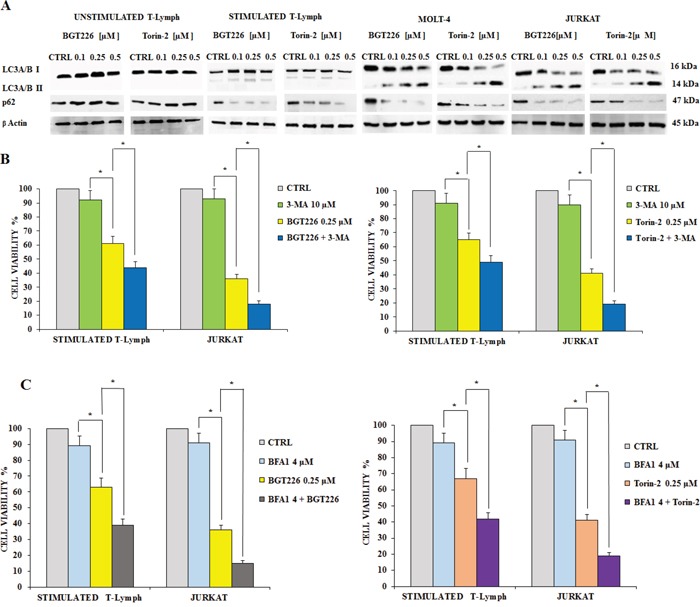
BGT226/Torin-2 induced autophagy in unstimulated, stimulated T lymphocytes and T-ALL cell lines **A.** Western blot analysis of primary T lymphocytes and T-ALL cell lines treated for 24 h with increasing concentrations of BGT226 and Torin-2. An increase of expression of fast-migrating (lipidated) LC3A/B and a reduction of p62 in stimulated T lymphocytes, MOLT-4 and JURKAT cells is shown. Twenty-five μg of protein were blotted on each lane. β-actin documented equal lane loading. **B.** MTT assay documenting the effect of the autophagy inhibitor 3-MA (3-Methyladenine) on the viability of stimulated T lymphocytes and JURKAT cells treated for 24 h with BGT226 and Torin-2. **C.** MTT assay documenting the effect of the autophagy inhibitor Bafilomycin A1 on the viability of stimulated T lymphocytes and JURKAT cells treated for 24 h with BGT226 and Torin-2. Results are the mean of three different experiments ± SD. Asterisks indicate significant differences with respect to untreated cells (*p< 0.05).

Autophagy plays a very important role in cell physiology, either as a form of cell death or as a protective mechanism against apoptosis [39, 40]. Therefore, there is a growing interest on the pharmacological approaches aimed to regulate autophagy, which represent a new area for the development of therapeutics protocols. Moreover, over the last few years various papers described the occurrence of autophagy in acute leukemia cells [reviewed in 42]. To find out if BGT226 and Torin-2 could induce autophagy, we analyzed the expression levels of microtubule-associated protein 1 light chain 3 LC3A/B I (non-lipidated form) and of its conjugated form LC3A/B II (lipidated). After 24 h of treatment with BGT226 and Torin-2, the unstimulated T lymphocytes showed no expression of LC3A/B II which, in contrast, increased gradually in a concentration dependent manner, for both drugs, especially in MOLT-4 and JURKAT cells (Figure 5A). We also analyzed the expression of p62, another marker of autophagy. p62 levels decreased in response to drug treatments in stimulated CD4^+^ lymphocytes and leukemic cell lines, but remained unchanged in quiescent T-cells (Figure 5A).

To establish whether autophagy was either a cell death or survival mechanism, we employed the autophagy inhibitor 3-Methyladenine (3-MA), which inhibits autophagy by blocking class III PI3K [41]. We also used Bafilomycin A1, another autophagy inhibitor, to further assess the mechanism of drug cytotoxicity. We treated stimulated T lymphocytes and JURKAT cells with BGT226, Torin-2, 3-MA or Bafilomycin A1 alone and in combination for 24 h. Results showed that 3-MA or Bafilomycin A1 alone did not affect cell viability, even at high concentrations (10 μM or 4 μM, respectively). On the other hand, when 3-MA or Bafilomycin A1 were administered with 0.25 μM BGT226 or Torin-2, the cells became more sensitive to the cytotoxic effect of both drugs (Figure 5B and 5C). These findings highlighted a protective role of autophagy from cytotoxicity induced by BGT226 and Torin-2 in stimulated T-lymphocytes and in JURKAT cell line.

### Apoptosis is required for mediating BGT226 and Torin-2 cytotoxicity

**Figure 6:**
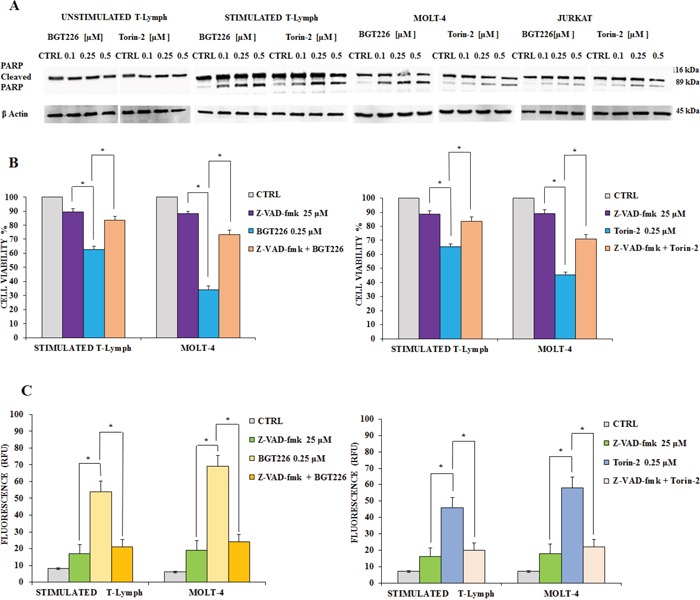
BGT226/Torin-2 induced autophagy in unstimulated, stimulated CD4^+^ T lymphocytes and T-ALL cell lines **A.** Western blot analysis documenting the increase of PARP cleavage in stimulated T lymphocytes and T-ALL cell lines treated for 24 h with increasing concentrations of BGT226 and Torin-2. Twenty-five μg of protein was blotted on each lane. β-actin served as loading control. **B.** MTT assays after BGT226 and Torin-2 treatment, alone and in combination with the pan caspase inhibitor z-VAD-fmk, in stimulated T lymphocytes and MOLT-4 cells. The analysis was performed after 24 h of treatment with BGT226 and Torin-2 at 0.25 μM and z-VAD-fmk at 25 μM. **C.** enzymatic cleavage of the profluorescent substrate Z-DEVD-R110, with release of the intensely fluorescent rhodamine 110-cleaving group, after BGT226 and Torin-2 treatment, alone and in combination with the pan caspase inhibitor z-VAD-fmk, in stimulated T lymphocytes and MOLT-4 cells. The analysis was performed after 24 h of treatment with BGT226 and Torin-2 at 0.25 μM and z-VAD-fmk at 25 μM. Results are the mean of three different experiments ± SD. Asterisks indicate significant differences with respect to untreated cells (*p< 0.05).

Previous studies documented that in T-ALL cells BGT226 could induce apoptosis [42]. In addition, our recent results demonstrated that Torin-2 is effective in Pre-B precursor-ALL cells [43]. In order to establish whether decreased viability was due to apoptosis, unstimulated and stimulated T lymphocytes as well as MOLT4 and JURKAT cell lines, were incubated with increasing concentrations of BGT226 and Torin-2 for 24 h, then Western blot was performed for analyzing the expression levels of poly(ADP-ribose)polymerase (PARP). The unstimulated T lymphocytes displayed no evidence for apoptosis. In contrast, significant cleavage of PARP was observed, especially in stimulated T lymphocytes, but also in MOLT-4 and JURKAT cells (Figure 6A).

To elucidate whether caspases were involved in the apoptotic activity of BGT226 and Torin-2, we analyzed the effect of z-VAD-fmk, a broad-spectrum caspase inhibitor whose activity had been checked already in different cancer cells [44, 45]. We administrated z-VAD-fmk alone and in combination with BGT226 or Torin-2 for 24 h in stimulated T lymphocytes and MOLT-4 cells, then cells were analyzed by MTT assays. Results showed that z-VAD-fmk (25 μM) alone had no relevant effect on cell viability, however when combined with BGT226 and Torin-2, it significantly inhibited apoptosis mediated by both drugs, in stimulated T lymphocytes and MOLT-4 cells. Thus, these findings indicated that BGT226 and Torin-2 induced a caspase-dependent apoptosis (Figure 6B).

We also measured caspase 3/7 activation by enzymatic cleavage of the profluorescent substrate rhodamine 110, bis-N-CBZ-L-aspartyl-Lglutaml- L-valyl-L-aspartic acid amide (Z-DEVD-R110), with release of the intensely fluorescent rhodamine 110-cleaving group [46]. The activity of caspase 3/7 was increased after drug treatment and was down-modulated by z-VAD-fmk (Figure 6C).

**Figure 7:**
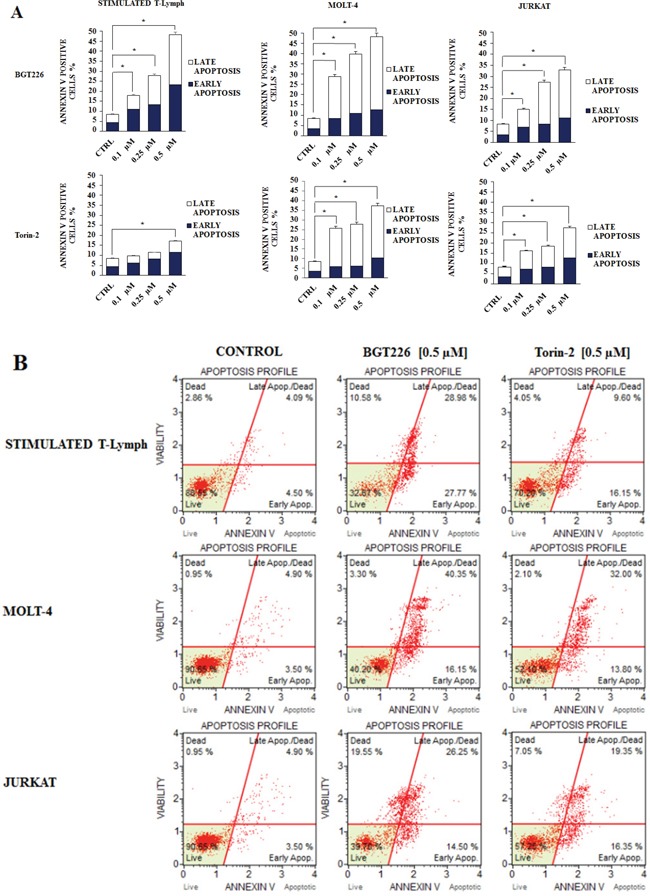
Flow cytometric analysis of drug-induced apoptosis **A.** Analysis of Annexin-V positive cells after BGT226 and Torin-2 treatment using the Muse™ Cell Analyzer in stimulated CD4^+^ T lymphocytes, MOLT-4 and JURKAT cells. The analysis was performed after 24 h of treatment with increasing concentrations of the drugs. Results are the mean of three different experiments ± SD. **B.** Flow cytometric plots of Annexin V-FITC/PI stimulated T lymphocytes, MOLT-4 and JURKAT cells, treated with 0.5 μM BGT226 and Torin-2. One representative of three different experiments that yielded similar results is shown.

Furthermore, we studied drug-induced apoptosis using Annexin-V staining in stimulated T lymphocytes, MOLT-4 and JURKAT cell lines treated with BGT226 or Torin-2 for 24 h. BGT226 effect was more relevant in MOLT-4 and stimulated T lymphocytes than in JURKAT cells, while the effect of Torin-2 was stronger in MOLT-4 and JURKAT cells than in stimulated T lymphocytes (Figure 7A). The percentages of live, early and late apoptotic cells in response to treatment with BGT226 and Torin-2 (0.1, 0.25 and 0.5 μM) are shown in Figure 7B.

## DISCUSSION

Although preclinical studies have demonstrated that inhibition of PI3K/Akt/mTOR axis could be an effective strategy for targeted therapy of T-ALL [47, 48], it is still unclear which is the best target in this highly complex and branched signaling network. Pharmaceutical companies have synthesized a wide array of drugs, that target different components of this signaling parthway [49, 50]. This allows to envisage different strategies based on one side on the use of selective inhibitors displaying reduced adverse effects, and on the other side pan-inhibitors expected to exert a greater efficacy.

It has been recently documented that PI3K inhibitors were the most effective in inhibiting leukemic cell proliferation and survival in a panel of T-ALL human cell lines, irrespectively of PTEN status. These findings strongly supporting clinical application of class I PI3K pan-inhibitors rather than dual γ/δ or single-isoform inhibitors for treatment of the majority of T-ALL patients [51].

Nevertheless, the functions of the immune system cells could be impaired by these drugs. Here we evaluated the effects on human healthy CD4^+^ T-cells of four PI3K/Akt/mTOR inhibitors used as anticancer agents, to explore how these treatments could affect the patient immunological status.

No cytotoxic effects were displayed by the drugs on unstimulated CD4^+^ T lymphocytes, whereas all the inhibitors and particularly BGT226 and Torin-2 reached IC_50_ values in the nanomolar range in T-ALL cells. In consideration of these findings, we performed the following sets of experiments using only BGT226 and Torin-2.

As to the cell cycle, since the unstimulated CD4^+^ T lymphocytes are mostly in a quiescent status, the drugs did not exert any effect, thus demonstrating the selectivity of the inhibitors.

In contrast, in either stimulated CD4^+^ T-lymphocytes or MOLT4 and JURKAT cell lines, BGT226 and Torin-2 induced a concentration-dependent accumulation in the G0/G1 phase, and a concomitant decrease in S and G2-M phases, as reported by others [52–54].

As shown by Western blot, Torin-2 and BGT226 effectively blocked the phosphorylation of Akt Ser 473 and Thr308, GSK3β Ser21/9, mTORC1 Ser2448 and mTORC2 Ser2481. Furthermore, an mTORC1 downstream target, S6 protein, was fully dephosphorylated in both stimulated T lymphocytes and T-ALL cells.

Healthy unstimulated CD4^+^ T lymphocytes did not display induction of autophagy as documented by Western blot analysis of LC3A/B. Stimulated T lymphocytes appeared more sensitive to induction of autophagy when compared to unstimulated ones. We also documented that in leukemic cells the induction of autophagy was a protective mechanism, since cells incubated with autophagy inhibitors were less viable. Similar results were obtained in stimulated T lymphocytes.

The drugs also induced apoptosis in both stimulated T lymphocytes and leukemic cells. On the contrary, resting T lymphocytes did not undergo apoptosis. The apoptotic cell death was dependent on caspase activity. Therefore, apoptosis is one of the mechanisms that explain the cytotoxicity of these drugs.

It is now assumed that tumor growth and survival can be either promoted or restrained by immune system cells [55, 56]. Therefore, it is critical to define whether novel anti-tumor drugs may impact on cells of the immune system. The ideal targeted therapy should specifically hit cancer cells and enhance anti-tumor immunity while preserving patient immunity [24].

It has been demonstrated that lymphocyte functions were minimally affected by p110α inhibition both *in vitro* and *in vivo*. Although p110α inhibition partially diminished Akt activation, it is likely that selective p110α inhibitors will be less immunosuppressive *in vivo* than p110δ or pan PI3K class I inhibitors [24].

Natural killer cell-mediated cytotoxicity as well as antibody dependent cellular cytotoxicity against tumor cells were significantly impaired by pan class I PI3K inhibitors, whereas p110α selective drugs had no effect [51, 57].

Other authors have shown recently that single inhibitors of class I PI3K isoforms in T-lymphocytes exerted a less potent impairment of T-cell activation than simultaneous inhibition of two or more isoforms [54]. These results suggest that complete blockade of class I PI3K activity strongly impairs T lymphocyte proliferation and activation *in vitro*.

In addition, it has been demonstrated that two ATP-competitive PI3K pan-class I inhibitors, PX-866 and BKM120, showed differences in their ability to block T-lymphocytes proliferation and IL-2 secretion [26].

Nevertheless, these investigations were restricted only to pan class I PI3K isoform inhibitors and did not explore other drugs, such as Torin-2 and BGT226, which target to additional components of the PI3K/Akt/mTOR axis.

Therefore our data expanded the concept that targeted therapies, using different drugs against molecules at different levels of the PI3K/Akt/mTOR cascade, may be effective against tumor cells harboring aberrant upregulation of this signaling network, without affecting at the same time the immune system.

Given the commonly observed dysregulation of PI3K/Akt/mTOR pathway in T-ALL, the various types of single or dual pathway inhibitors under development might be effective in T-ALL treatment, provided that they do not affect the immune system. Therefore, the study of the effects of PI3K/Akt/mTOR inhibition not only in tumor cells, but in immune cells as well, may lead to selection of treatments that, while efficiently targeting deregulated PI3K/Akt/mTOR signals in tumor cells, preserve normal immune function, for appropriately tuning of personalized cancer therapy.

## MATERIALS AND METHODS

### Materials

RPMI-1640 medium, fetal bovine serum (FBS), penicillin and streptomycin were purchased from Lonza Milano SRL (Milan, Italy). Torin-2, NVP-BGT226, MK-2206 and ZSTK474 were obtained from Selleck Chemicals (Houston, TX, USA). For cell viability determination, Cell Proliferation Kit I (MTT) was purchased from Roche Applied Science (Basel, Switzerland). Annexin V/7-ADD detection kit and cell cycle kits were from Merck-Millipore (Darmstadt, Germany). For Western blot, antibodies to total Akt-1, Ser473 p-Akt-1 and Thr308 p-Akt-1 were from Santa Cruz Biotechnology (Santa Cruz, CA, USA), while all the other antibodies were from Cell Signaling Technology (Danvers, MA, USA), including the rabbit secondary antibody. The mouse secondary antibody, z-VAD-fmk, 3-Methyladenine (3- MA), Bafilomycin A1, Ficoll-Paque Plus and phytohemagglutinin were purchased from Sigma Aldrich (Milan, Italy). Dynabeads T-cell separation kit was from Invitrogen life Technologies (Monza MB, Italy). Signals were detected using ECL Plus reagent from Perkin Elmer (Boston, MA, USA).

### Cell culture and Western blot analysis

#### Cell lines

T-acute lymphoblastic leukemia cell lines obtained from Deutsche Sammlung von Mikroorganismen und Zellkulturen GmbH (Braunschweig, Germany). JURKAT and MOLT-4 were maintained in RPMI-1640 medium supplemented with 10% heat-inactivated fetal bovine serum (FBS), 100 units/ml penicillin and 100 mg/ml streptomycin at a density of 0.5 to 2 × 10^6^ cells/ml and were incubated at 37°C with 5% CO_2_.

#### Primary samples

Peripheral Blood CD4+ T lymphocytes from healthy donors were obtained with informed consent according to institutional guidelines and isolated with Ficoll-Paque and magnetic beads labelling protocols (Dynabeads, Monza MI, Italy). Whole blood or buffy coat were diluted with PBS containing 0.1% BSA and 0.6% Na-citrate or 2 mM EDTA (without Ca2+ and Mg2+) in ratio 1:1. Thirty-five ml of the diluted sample were layered over 15 ml of Ficoll-Paque medium and centrifuged at 600 g for 40 minutes at 20°C. The peripheral blood mononuclear cell (PMNC) layer was transferred to centrifuge tube containing three volumes of PBS and centrifuged at 100 g for 10 minutes at 20°C. This step was repeated twice, supernatant was discarded and cells were suspended in complete RPMI-1640 medium.

500 μl PMNC were transferred into a test tube, at a density of 5 × 10^7^ cells/ml supplemented with100 μl of heat inactivated FBS and antibody mix and incubated at 4°C for 20 minutes. Followed by addition of 4 ml isolation buffer and centrifuged at 350 g at 4°C for 8 minutes. The supernatant discarded, and the pelleted cells were suspended in 500 μl of isolation buffer, added with 500 μl of pre-warmed dynabeads and incubated for 15 min at 20°C. The cells bound to beads were resuspended using 4 ml isolation buffer. The supernatant containing the human CD4^+^ T lymphocytes was obtained by placing the resuspended cells in magnet for 2 minutes. The human CD4^+^ T lymphocytes were grown in complete RPMI-1640 medium with 10μg/ml phytohemagglutinin at a density of 1 × 10^6^ cells/ml, in a CO_2_ incubator at 37°C for 24 h [58, 59].

#### Western blot

The cells were homogenized for 30 min in cold lysis buffer (50 mM Hepes pH 7.5, 5 mM EDTA pH 8.0, 10 mM MgCl2, 150 mM NaCl, 50 mM NaF, 20 mM β-glicerophosphate, 0.5% NP40, 0.1 mM sodium orthovanadate, 1 mM PMSF, 1 mM DTT) containing the protease inhibitor cocktail obtained from Roche Applied Science Basel, Switzerland. Lysates were centrifuged in a Microfuge for 10 min at 4°C and 25 μg of solubilized proteins were resolved by 10% or 12% SDS-PAGE [60, 61].

### Cell viability analysis

MTT (3-[4,5-Dimethylthythiazol-2-yl]-2,5-Diphenyltetrazolium Bromide) assays were performed to assess the sensitivity of cells to drugs, as described [62, 63].

### Cell cycle and apoptosis analysis

Cell cycle analysis was performed using the Muse™ Cell Analyzer (Merck Millipore, Milan, Italy). Cells were harvested after 24 h of treatment with the drugs, centrifuged at 300 g for 5 minutes and washed with 1x PBS. The cells were then fixed with 70% cold ethanol for 3h at −20°C, centrifuged at 300 g for 5 min and washed with 1x PBS. Each sample was then resuspended in 200 μl of Muse™ Cell Cycle reagent, incubated in the dark for 30 min at room temperature, and analyzed according to the manufacturer's instructions. The analysis of apoptosis was performed by Annexin V/7-ADD-Assay. The cells treated with increasing concentrations of BGT226 or Torin-2 were harvested after 24 h. The cell suspension was labeled in the dark for 20 min with an equal volume (100 μl) of the Muse™ Annexin-V Dead cell reagent (Merck Millipore). Subsequently, quantitative detection of Annexin-V/7-AAD positive cells was performed using the Muse™ Cell Analyzer.

### Caspase-3/−7 activity assay

Caspase activity was measured with the Apo-One Homogeneous Caspase 3/7 assay kit (Promega Corporation, Madison, WI, USA), according to the manufacturer's instructions. The induction of apoptosis and associated activation of caspases 3 and 7 were measured by enzymatic cleavage of the profluorescent substrate rhodamine 110, bis-N-CBZ-L-aspartyl-Lglutaml- L-valyl-L-aspartic acid amide (Z-DEVD-R110), which releases the intensely fluorescent rhodamine 110-cleaving group. Cells were seeded at a density of 1×10^5^/ml and incubated in a 96-well plate in the presence or absence of drug for 48 h. For cells treated with a combination of z-VAD-fmk and drugs, they were initially treated with z-VAD-fmk for 4 h prior to the addition of drugs. 100 μl of the homogeneous caspase-3/−7 reagent was added to each well and the reaction mixture was incubated for 2 h at room temperature. Fluorescence was measured at an excitation wavelength of 485 nm and an emission wavelength of 538 nm. Results are expressed as relative fluorescence units (RFU).

### Statistical evaluation

The data are presented as mean values from three separate experiments ± SD. Data were statistically analyzed by a Dunnet test after one-way analysis of variance (ANOVA) at a level of significance of P<0.05 vs control samples [64].
